# It changes your mind: doing gender by proxy and gender fluidity in the context of dementia

**DOI:** 10.3389/fpubh.2026.1931315

**Published:** 2026-09-29

**Authors:** Jordana L. Clayton

**Affiliations:** Department of Sociology and Criminology, University of Utah, Salt Lake City, UT, United States

**Keywords:** aging, bioethics, cognition, dementia, gender, sex, surrogate decision making

## Abstract

People with dementia progressively lose the ability to act and make decisions independently, requiring a prolonged period of supported decision making and possibly complete decision making by proxy in the final stages of the disease. Sometimes, the decision-making process is complicated by the fact that people with dementia have different personalities, preferences, desires, and values compared to earlier in their lives, creating a conflict between earlier values and preferences and currently expressed ones. Most examinations of this process examine caregivers’ proxy decisions around medical care, finances, and legal matters. However, caregivers engage in supported and proxy decision making processes in multiple areas of life, such as activities, dress, grooming, overall lifestyle; these areas of life are profoundly gendered for most people. This paper will extend the discussion of supported and proxy decision using West and Zimmerman’s (1987) concept of doing gender and Judith Butler’s (1990) gender performativity theory to theoretically examine how caregivers do gender by proxy for people with dementia and the possible impacts of having changing values, preferences, and desires around gender for people with dementia. First, I will review sociological theories of doing gender and gender performativity, followed by a review of current bioethical concepts of proxy decision making and the conflict between changing values and preferences prior to and during dementia. Then I will combine these theoretical traditions to conceptually define doing gender by proxy and gender-related supported decision making both in community and institutional settings.

## 1 Introduction

Dementia is a neurocognitive illness that leads to changes in behavior, personality, and decision-making (1, 2). People with dementia progressively lose the ability to act and decide independently and may require supported decision making or decisions to be made by a proxy / surrogate decision maker (3–7). Caregivers play a substantial role throughout these changes in supporting a person with dementia in maintaining their quality of life, sense of self, and autonomy (3, 4, 8, 9). While literature on this subject focuses primarily on medical, financial, and legal decisions (4, 10), caregivers provide supported and proxy decision making in many other areas as well, such as dress, grooming, hobbies, household tasks, and social activities. These areas of life are profoundly gendered and contribute to the maintenance of gender identity for people with dementia, just like they do for most everyone else (9, 11).

People with dementia often experience personality and behavioral changes and may have different values, preferences, and feelings about their gender presentation and / or identity compared to earlier in their life (9, 12). While this could be perceived pathologically, as non-normative expressions of gender often are, I join previous researchers in advocating for a positive and affirming approach that centers the dignity and autonomy of people with dementia and their right to use gender expression as a way to maintain personhood and identity (9, 12, 13).

This paper uses sociological theories of doing gender (14) and gender performativity (15) to formally conceptualize and define doing gender by proxy and explore the possible ramifications of changes in gender preferences, values, desires, and behaviors in people with dementia. I developed the concept of doing gender by proxy when a study of dementia-focused end-of-life care planning, supported and surrogate decision making, and dementia-induced changes in identity coincided with reading West and Zimmerman’s *Doing Gender* (14) and Judith Butler’s *Gender Trouble* (15). Pondering the connections between these two disparate theoretical traditions—doing gender / gender performativity and the bioethics of surrogate decision-making—led to the present analysis: a review of relevant literature (which does not rise to the level of a systematic review) and a synthesis of ideas culminating in a formalized definition of doing gender by proxy.

To my knowledge, this is the first conceptualization of doing gender by proxy and is among few studies that explore changing preferences and values on gender during the course of dementia. Gender performance and the gender experiences of older people remain underexplored in the literature, particularly in the context of dementia, with some notable exceptions (9, 11, 12, 16–21). All older adults have experiences with doing gender and all older adults have some component of their identity which involves gender (even if that is a lack of gender identity). “Gender is dynamic across the life-course for all people” (22). While dementia is a particularly useful tool for examining how personal transformations change or do not change gender performance and / or identity, I hope it becomes clear to the reader that these debates and perspectives apply to and are relevant to all of us.

## 2 Gender and performativity theory

### 2.1 Core concepts

Gender is a way of understanding, framing, and creating personal identity and social relationships. It is central to many people’s identity, and this happens in a feedback loop with others’ affirmations of one’s gender identity (15, 23). Gender fundamentally structures social life at both the macro and micro levels and across the life course (22, 24, 25). Early feminist conceptions of sex and gender emphasized that sex is the biological aspects of a person and gender is the socialized aspects of a person; sex describes your hormones and genitals, and gender describes the way you dress, groom yourself, and interact with others (14, 15, 26, 27). This allows for a “radical discontinuity between sexed bodies and culturally constructed genders” (15, p6) in which no particular gender can be assumed to follow from a particular sex, nor that the number of genders and the number of sexes must correspond to each other. This discontinuity provides an explanation for both historical and modern gender diversity (15, 28). Conceptually, a complete separation of sex and gender would be completely satisfactory if sex was not itself socially constructed in gendered ways; bodies are always interpreted through a gendered lens (29–31). However, for the purposes of this article, using sex to refer to biological characteristics and gender as behavioral characteristics can provide a useful basis for discussing possible changes in gendered behavior relevant to people with dementia, such as those in dress and grooming. Furthermore, since bodies of diverse characteristics are typically categorized as being either male or female, this paper will use West and Zimmerman’s (14) concept of sex category to reference a person’s social assignment as male or female.

Gender performativity theory is a way of understanding gender as being a social performance comprised of a continuous series of embodied acts that communicate gender (e.g., man, woman, nonbinary) to others using actions like dress, grooming, and social behavior (14, 15, 32). This theoretical tradition springs from earlier sociological theories of performance, such as Goffman’s conceptualization of the self as the combination of a character performed for a social audience and the performer who chooses what act to put on (23, 33, 34). Performers iteratively choose their performance based on the character one seeks to create and the audience response to that character, i.e., social reactions (23, 33).

West and Zimmerman (14) made a landmark contribution by describing gendered social performativity as “doing gender,” in which people perform actions that are perceived to be appropriate based on their sex category. Shortly after the publication of *Doing Gender* (14), Judith Butler made a similarly groundbreaking contribution with *Gender Trouble* (15). West and Zimmerman (14) approach doing gender as an interactive phenomenon in which people are socially accountable for a performance based on their sex category, while Butler’s (15) theory focuses on iterative and reiterative production of gender under a regulatory heterosexual framework. Both traditions emphasize that gender performances are not central to any difference in biology between males and females; the social organization which causes women to be primarily responsible for cooking and cleaning while men are primarily responsible for automotive care has nothing to do with chromosomes or genital configuration. However, many social behaviors interactively become gendered. A combination of personal desire to align behavior with one’s gender identity and social expectations (and punishments) coerce people to do their gender ‘correctly’ or face the consequences. This creates a combination of opportunities and constraints for people within each sex category. Performing gender can provide immense satisfaction, enjoyment, and sense of ‘being oneself’ but can also create strain and difficulty when gender expectations do not align with a person’s desired actions or identity.

### 2.2 Doing gender and identity

Within performativity theory, the external and embodied acts of the performer create the sense of a coherent, internal identity, not the other way around (23, 33). Rather than assuming that gendered behaviors emerge from an innately gendered core, performativity theory argues that consistently engaging in gendered behaviors is what makes a person ‘feel’ their gender identity. Within this understanding, “gender is not something we are, but something we do” (35). Butler argues that people desire and idealize internal coherence and wholeness, and organize their performances to sustain the sense of having a complete internal core identity, including gender identity, through a production that occurs “on the surface of the body” (15, p136) such as grooming, dress, gestures, and speech. Engaging in these performative acts is learned from a very young age and as acts are repeated consistently over time, they create the sense of a stable self (14, 35). Inability to perform gendered acts can threaten the stability and coherence of gender identity (14, 15, 36).

Gender is not the only social identity that is performed; performativity theory was originally developed as a theory of personality and social behavior (23, 33, 34). People also “do” race, class, age, nationality, parenthood, religion, and other social identities, and the combination of multiple social identities creates different patterns of “doing” among groups and individuals (37–39).

### 2.3 Structural constraints in doing gender

While the performer has the opportunity to choose their performance, they are unable to choose the social interpretation of their performance or the consequences of choosing a socially undesirable performance (14, 33). Gender is a social structure, like race and class, meaning that while almost everyone in society participates in and influences the structure, no single person is able to control the structure as a whole or the power dynamics and ideology that motivate the structure (24, 25). West and Zimmerman “contend that the ‘doing’ of gender is undertaken by women and men whose competence as members of society is hostage to its production” (14). Failing to appropriately perform gender as expected based on sex category and other social identity characteristics (race, class, parenthood, nationality, marital status, religion) typically causes social punishment of various kinds (14, 15, 40). These punishments can range from mild to extreme: a male wearing a dress on public transit may receive odd looks, verbal slurs, harassment, or may be assaulted or murdered, depending on the reactions of others.

Butler argues that people who fall outside of gendered understandings are dehumanized. Most social interactions are intricately scripted based on gender—typically the interaction of the genders of the people involved—and therefore social scripts fail to provide guidance on how to treat people without an intelligible gender. Lacking an appropriate social script, or a pre-determined pattern of gendered performance, can cause social anxiety, resentment, and dehumanization (15, 23, 33, 34, 41, 42). “Those bodily figures who do not fit into either gender fall outside the human, indeed, constitute the domain of the dehumanized and the abject against which the human itself is constituted” (15, p111).

### 2.4 Fluidity and flexibility in doing gender

Because gender is something that is constructed and performed continuously, it is dynamic over the life course and interacts with other social identities like race, class, and age (43, 44). As people age, their relationship with their gender changes as they acquire new social locations and are perceived differently by others (43). By emphasizing the instability and illusory nature of a core identity, performativity theory makes room for fluidity in identity over the life course. Social characteristics like gender, race, and sexual orientation are typically perceived as being fixed and unchangeable; in practice, this is not the case (22, 45–48). In the most direct sense, people can and do completely change their identified gender, race, and sexual orientation over the life course (31, 45, 46, 49, 50). There is also a more subtle version of fluidity, however, in which the way that these characteristics are performed and the relationship of the performer to their identity changes over time (22, 43, 44, 46). As described by Brady et al., “Gender is dynamic across the life-course for all people. … we invite you to reflect on how changes in the experience and expression of gender can be most appropriately viewed as a normal part of human development, diversity, and growth. … one’s own gender and relationship to it can change, evolve, weaken, and galvanize across a lifetime” (22). Rather than being fixed, gender is flexible through changes in performance and meaning-making by the performer.

## 3 Bioethics in the context of dementia

### 3.1 Ethical standards for decision making by proxy

Dementia causes progressive cognitive impairment that makes independent reasoning, judgment, and decision making increasingly difficult over time (1, 5). Because of this, people with dementia eventually may require a proxy decision maker to make necessary legal, medical, and financial decisions entirely on their behalf (3, 5). This paper will use the terms surrogate decision maker / making, proxy decision maker / making, and decision making by proxy interchangeably, as they all refer to the same concept. Surrogate decision making can be distinguished from supported decision making, in which a person with dementia is assisted in making decisions for themself and being involved in decisions about themself for as long as possible (51, 52). Decision making can be viewed as a spectrum, with completely autonomous decision making on one end, supported decision making in the middle, and surrogate decision making on the other end. Decision-making competence can fluctuate with time and can be decision-specific; a person with dementia may be unable to independently decide on complex financial decisions but entirely capable of deciding how to spend their afternoon (53).

When a person with dementia requires a surrogate decision maker, there are three traditional decision-making standards theoretically followed in a hierarchy (3). The first is to follow the person’s known wishes, in which the person describes their preferences (typically prior to losing decision-making capacity) and these are enacted as exactly as possible. This is widely considered the gold standard of surrogate decision making. If the person’s wishes are not known, the second standard is substituted judgment, in which the surrogate decision maker uses the person’s known values to determine which option they would most likely select if they could. If the person’s values are also unknown, then the final standard is to make decisions in the best interest of the person as determined by their surrogate decision maker and clinical care team. In practice, surrogate decision making often does not follow these cleanly-cut standards because advance directives / living wills lack specificity, people tend to be more concerned with their surrogate’s ability to dynamically respond to new information than with their ability to rigidly follow a predetermined set of values, and medical decision making is typically more complex than what can be prepared for through documentation or discussion (3). Furthermore, people with dementia are different from other people requiring surrogate decision-making (such as people in a coma) because they remain an active participant in decision-making processes throughout the disease course, even when they need supported decision making or cannot independently decide (4, 53).

A person with dementia should remain involved in the decision-making process as long as possible (51–53). Maintaining involvement in decision-making is central to preserving a person with dementia’s dignity and autonomy (4, 8, 54). While some understandings of personal autonomy are highly individualistic, decisions for end-of-life care are often experienced as a social and relational process in which trust, mutual support, and shared discussion forms the basis of the decision-making process; this has been labeled as relational autonomy (54, 55). Relational autonomy is especially important for people with dementia who must trust others to enact their wishes and protect their wellbeing. Because people with dementia lose the ability to act completely independently, they are vulnerable to abuse, neglect, and coercion; those with decision-making authority for / over them have a particular responsibility to prioritize their current desires and best interests (56).

### 3.2 Quality of life and decision making for a person with dementia

What is in the best interest of a person with dementia can be difficult to judge, and typically comes down to preserving a combination of quality of life, autonomy, and dignity. Like other disabilities, life with dementia is socially constructed as highly degraded and characterized by undignified suffering (56). Disability rights advocates emphasize that many disabled people find life worthwhile, enjoyable, and of high quality, even when experiencing pain and limitations that other people perceive as intolerable (56–58). While the disability itself may cause difficulties, a substantial portion of the suffering experienced by people with dementia and other disabilities is from social discrimination and a lack of accommodations which prevent disabled people from being integrated into society (56). Many people with dementia adapt to their functional and cognitive limitations and find new ways to experience and enjoy life (56, 59). “You adjust to things you never thought you could—not driving, losing your independence” (56). However, most people prepare advanced directives and share their wishes for their end-of-life care before diagnosis or early after diagnosis when they have not yet had time to discover the extent to which they may adjust to life with dementia (4). This can create a conflict between previously held ideas about life with dementia and lived experience which leads to uncertainty for surrogate decision makers about which preferences to follow when making end-of-life care choices.

The classic case put forward to highlight the difficulties created by this conundrum is the story of Margo, a happy old woman with dementia (10, 60–62). Prior to having dementia, Margo was an energetic and intelligent woman who had a clearly stated preference to not live with advanced dementia. If she were to develop dementia, she did not want to be treated for any health problems that arose, and wanted her money to be donated to charity rather than being used for her care (10). Later on, Margo did develop dementia, during which she:

derive[d] obvious satisfaction from simple pleasures such as eating peanut butter and jelly sandwiches, reading and rereading mystery novels, listening to songs over and over, and attending an art therapy group where she paints the same picture every week. While she previously would have regarded such an existence with repugnance, she now appears quite content in it.

If Margo were to develop pneumonia, prevailing ethical standards would require us to withhold antibiotics out of respect for her previously expressed values, despite her present interest in surviving a treatable infection and continuing a life that she now finds agreeable (10).

In alignment with the value that Margo should remain involved in decisions about her own treatment whenever possible, supported decision making practices could provide an accommodated explanation of the treatment and its possible side effects (52, 56). If this were not possible, a surrogate decision maker would need to weigh Margo’s earlier preferences against her current preferences to make the decision.

### 3.3 Conflicts between previous and current preferences and the autonomous ‘true self’

Margo’s story highlights that behind the practical dilemma of deciding whether or not to treat an infection or other health problem lies a philosophical debate about the nature of personal identity. Most directly involved is the question of whether a person with dementia remains essentially the same person or becomes a different person due to their change in personality, values, and judgment. Following this, if the person with dementia is judged to be the same person but has changing values and preferences, the question arises as to whether the original, pre-dementia values and preferences should be prioritized over the values and preferences they express during the course of their disease.

Before specific arguments for or against a person with dementia remaining the same person, it will be useful to examine basic assumptions about what constitutes sameness. As described by Parfit (63), philosophers distinguish between qualitative and numerical sameness. Qualitative sameness is when two things have the same qualities, such as two monarch butterflies with identical patterning. Numerical sameness is the persistence of the same object over time, even with qualitative changes. A monarch caterpillar that transforms into a butterfly is qualitatively different but numerically the same. Appeals to each of these kinds of sameness are used in arguments about changing or unchanging personal identity.

Parfit (63) argues that a self is created by a chain of connected selves and memory is central to maintaining connections in that chain. Because of that, once the chain is severed by inability to remember previous events, then the self becomes a different person. Parfit (63) relies on a qualitative similarity of memory and psychological states as foundational to selfhood, regardless of the state of the body. Becoming a qualitatively different person severs the connection to the former self and makes the preferences of that other, former person essentially irrelevant to the life and treatment decisions of the current person.

On the other side of the debate, Dworkin (61) argues that a person with dementia remains the same person even if they are qualitatively different (a numerical sameness argument), and that what is lost in dementia are the critical interests that guide our values and reasoning, such as the interest in dignity or in philanthropy. What is left in dementia is the experiential interest, such as the interest in eating peanut butter and jelly sandwiches and listening to music. Dworkin (61) argues that critical interests are what ought to be preserved and respected when making end of life decisions, because these guide the creation of a life story and give life meaning. This perspective has been criticized, notably by Dresser (61), who argues against the preferential treatment of critical interests because a good quality of life can be maintained even when only considering experiential interests.

Outside the realm of academic bioethics, Earp et al. (64) ran surveys to determine what the typical perspective is among non-academics as to whether a person with dementia remains the same person. Their team also gauged popular views on whether an advance directive from a time prior to dementia should take precedence over the current preferences of the person with dementia. Earp et al. (64) provided a vignette similar to the story of Margo about a happy older woman with dementia named Robin; half of participants were randomly assigned to also receive information that Robin had made an advanced directive prior to dementia requesting to receive no treatment if she had lost mental capacity. Less than half of participants judged that Robin was still her true self, although this percentage was higher in the group not given information about an advanced directive (40.9%) than in the group that read about the advanced directive (28.8%). More than half of participants in both groups judged that Robin should be treated for pneumonia, though the group with no information about an advanced directive were much more likely to be in favor of treatment (85.8%) than the group given information about the advanced directive (52.4%). Despite the fact that just over 70% of both groups believed that previous preferences should determine treatment, in practice many participants in the advanced directive group did not choose to follow Robin’s previously stated preferences.

Taken together, this research indicates that there is no popular consensus on the nature of the self, on the importance of preferences given prior to dementia, or on how to make decisions in practice for someone with dementia to maximize their current well-being. It is unsurprising, then, that surrogate decision makers struggle to decide and do not consistently use any particular standard of surrogate decision-making (3–5).

### 3.4 The mind–body split and the (un?)gendered ‘true self’

Western theories of the self emphasize thought, rationality, and cognitive function, most famously articulated by René Descartes as “I think, therefore I am” (9, 65). Descartes’ philosophy gave rise to what is now known as the Cartesian mind–body split or mind–body dualism, in which the mind and body are considered to be separate entities and only the mind is essential to personhood, not the body (65, 66). Consider the following passage from Descartes:

… I find that thought is an attribute which belongs to me; it alone cannot be separated from me. *I am, I exist, —*that is certain, but for how long? As long as I think; for, perhaps, if I entirely ceased to think, I should at the same time entirely cease to be. I now admit nothing which is not of necessity true; therefore, strictly speaking, I am *only a thing which thinks*, that is to say, a mind, an understanding, or a reason … I am not this collection of members which is called the human body … (65).

In this conceptualization of personhood, the body is unnecessary to personhood. People with cognitive impairments or even ‘irrational’ thoughts are cast as less of a person than those with ‘complete’ or ‘superior’ cognition (9). Person-centered approaches to dementia, initially developed by Kitwood (67) and Kitwood and Bredin (68), have attempted to center the humanness and personhood of people with dementia, but these approaches are fighting centuries of social conditioning in which the rationality of the mind is what makes a person a person (9, 69, 70).

Although Descartes originally argued that the mind is sexless and unaffected by bodily differences, he also argued that men possess greater reason and intellect than women (65, 71). Within philosophy and popular perception, the mind, reason, and rationality came to be closely associated with maleness and masculinity, while the body and embodiment became associated with femaleness and femininity, particularly through reproduction (15, 66, 72). “Women are somehow *more* biological, *more* corporeal, and *more* natural than men” (73). Similarly, the mind was associated with activity and culture while the body was associated with passivity and nature (15, 72), creating two sets of connected concepts: masculinity / maleness / the mind / reason / activity / culture superior to and acting on femininity / femaleness / the body / feeling / passivity / nature (15, 72). Feminist theories of sex, gender, and embodiment have challenged binarist philosophies that focus on difference and separation rather than similarity and wholeness (15, 72, 74). This has included challenging the concept of the mind–body split and the relative unimportance of the body compared to the mind (74).

Dementia causes a person who was formerly rational, cultured, and active (all masculine-coded traits) to become emotional, embodied, and passive (feminine-coded traits) and thus debased and inferior. This is reflected in bioethical discussions of dementia. Dworkin’s (61) delineation of critical interests being more ethically relevant than experiential interests reflects this divide: personal interests that are guided by reason are privileged over those that are guided by embodied pleasure. Criticism of this perspective argues against the superiority of the mind and in favor of embodied ways of maintaining selfhood. As perfectly encompassed by Ward, Campbell, and Keady:

Notions of personhood and accompanying models of care have, in the past, been unhelpfully ‘disembodied’ often meaning that the body has remained the exclusive domain of medical science. As a result, much of the debate on dementia has overlooked the embodied dimension to self and identity, and neglected the simple fact that our bodies are the basis on which we engage with the world. In short, we have underplayed the role of the body in the agency, self-expression and relationships of people with dementia (75).

Sex and gender are core parts of embodiment and embodied identity (9). Thus, maintaining gendered embodiment is a way that people with dementia can access and maintain their relationship to agency, autonomy, and personal identity.

## 4 Gender and dementia

Examining gender performance in the context of changing cognitive ability highlights the highly contingent nature of gendered performances on continuous maintenance and repetition, and reveals the extent to which gender is a consciously chosen, intentionally maintained personal characteristic rather than purely innate and fixed (14, 15, 35). The loss of control over the body and mind caused by dementia has been linked to a loss of ability to perform gender according to social norms and in a way that can be intelligible to others (9). This “de-gendering” (9) can become part of the loss of personhood and personal identity for the person with dementia, and fits into the overall social narrative in which dementia causes irreversible loss, decline, and personal devastation. Person-centered approaches to dementia push back on the narrative of pathology and devastation, instead trying to conceptualize of the person with dementia as a whole person (not just their disease) and maintaining a focus on quality of life (67–69).

### 4.1 Defining doing gender by proxy

Synthesizing concepts from gender performativity theory and bioethics, I offer the following definition: doing gender by proxy occurs when a caregiver makes or enacts gender-related decisions on behalf of a person with dementia who is unable to make that specific decision independently. Supported gender-related decision-making is conceptually related but distinct, occurring when the person retains decision-making capacity regarding gender-related choices but requires some assistance. Doing gender by proxy and supported gender-related decision making encompass gendered appearance and grooming, ways of talking to or about the person, what gendered social groups and activities the person is or is not included in, and other actions which create the sense of a gendered self for the person with dementia or maintain a gender performance for the benefit of others. Gender decisions should be firmly grounded in the will and preferences of the person with dementia in a way that honors their social relationships. Unfortunately, just as family members may coerce a person with dementia to pursue unwanted—or forgo wanted—medical treatment because of the desires of others, doing gender by proxy may involve the imposition of normative gendered behavior onto a person who wishes to act otherwise.

In a positive sense, caregivers (formal and informal) of people with dementia may attempt to help retain a sense of personhood and embodied selfhood through maintaining desirable gendered expressions, such as hair and dress (9, 75). Maintaining the personal appearance of people with dementia has been long assumed to be a care task that will be automatically taken up by caregivers, without significant thought as to the meaning making associated with it beyond that required by basic dignity (75). Despite this fact, appearance has been shown to be deeply meaningful for people with dementia and their caregivers as a way to express personal identity and to show care (75). Although appearance is not the only way to maintain a gendered self, it is a key part of gendered embodiment and personal autonomy.

Autonomy and the ability to choose is central to all decision-making processes, including and maybe especially for people with dementia. Whether sex and gender are or are not within the realm of personal choice / autonomy is currently under extreme social, political, religious, medical, and philosophical debate (76). Positioning agency as central to gender is not only an argument in favor of the legitimate ability to make autonomous decisions about one’s own sex and gender, but also aligns with prevailing bioethical norms in which bodily autonomy and the ability to choose are central to the maintenance of identity, personhood, and dignity (4, 8, 54, 77). Bioethical theories of surrogate decision making are therefore applicable to the process of making gendered choices for someone who can no longer independently decide and whose preferences may or may not match those from earlier in their life. As caregivers support the decision making of people with dementia, they will inevitably be faced with decisions that are gendered and relate to the gender values, preferences, and desires of the person with dementia. Because gender is so central to personal identity, it deserves to be an area in which careful thought is given to how to respect and maintain the autonomy of older adults with dementia as they begin to require others to offer supported decision making and eventually do gender by proxy for them.

Changes in the clothing or grooming that is desired by a person with dementia may not be indicative of new feelings about their gender or a new gender identity. Practical concerns can motivate changes in appearance, such as preferring clothes that are comfortable rather than stylish or cutting long hair so it is easier to wash (9, 75). Caregivers can support choices about appearance, dress, and grooming that prioritize both the emotional and physical comfort and desires of a person with dementia and take time to discuss the motivations for changes in gendered aspects of presentation.

### 4.2 Gender by proxy in community and informal caregiving

Considering informal care arrangements, Calasanti and Bowen (11) explicitly describe doing gender by proxy in their article on spousal caregiving and the maintenance of gendered identities within dementia. They note how “spousal caregivers work to maintain their spouses as men and women, and not simply as genderless ‘people.’ In this sense, caregivers play an integral role in helping their spouses accomplish or ‘do gender’” (11). They reported that both husbands and wives maintained their spouse’s gender through 1) maintenance of a gendered appearance through dress, grooming, and personal care and 2) purposefully arranging activities and behaviors to help their spouse maintain a gendered sense of self. Without using the language explicitly, Calasanti and Bowen (11) were describing spousal caregivers doing gender by proxy. What may be called gender care is a special type of invisible care work that acknowledges the centrality of gender performance to the maintenance of personhood and identity. Spouses had to learn “what the other person needed, in relation to gender; what women ‘need’ for instance, to be *women*. To the extent that spouses did this—and there was variation—they had to also learn to think in terms of a different gender identity” (11).

In addition to doing gender by proxy, caregivers may engage in supported decision-making in gender presentation for people with dementia. Consider the situation of Diane Norelius, featured in a 2023 New York Times article by Katie Engelhart (78). Most of the news article is about Diane’s daughters fighting Diane over her choice to leave a substantial portion of her inheritance to her new partner Denzil, but a few sentences describe a change in Diane’s gender presentation:

Soon, she started dressing differently. Before, Diane had lived in colorful blouses from Chico’s and swinging pendant necklaces. Now she dressed like a cowboy, in stiff snap-button shirts tucked into bluejeans, even though she had never tucked her shirts into her pants because she thought it made her look fat. She wore big leather belts too, and Denzil’s orange baseball cap. And sometimes the same outfit, day after day (78).

Changes in gender presentation accompanied other changes in personality, judgment, and decision-making for Diane. Notably, she exchanged her feminine blouses and necklaces for a masculine “cowboy” look. This is introduced in the article as part of the pathology of dementia and an indication that Diane was slowly losing the connection to her true self. A more expansive interpretation could see this as a fluid change in Diane’s gender presentation. Diane’s partner Denzil supported her change in presentation, providing her with his clothes to wear and supporting her in choosing a more masculine personal style (78).

Conceptualizing family members’ decisions to enact gender by proxy and engage in supported decision making around gender is part of the overall process of understanding how caregivers maintain the identity and personhood of the person with dementia (9). However, just as with medical care, balancing respect for previously held wishes of a person with dementia and new values and preferences may be difficult. For example, people who prior to dementia strongly identify with and prize their gender may find it extremely objectionable to lose that connection to their gender during dementia (9, 12). Thus, it may be difficult for caregivers to know the extent to which they should try to enact the person’s previous wishes versus their current desires and practical needs.

The decision of current versus previous preferences may be particularly thorny for caregivers of queer, transgender, nonbinary, and gender non-conforming people who may be afraid in unique ways to lose connection to their chosen gender presentation and identity during the course of dementia (21, 79, 80). Having changes in desired gender presentation be dismissed as part of the disease, as confusion, or as an additional pathology is also a particular concern (9, 12, 13, 19, 80). Complicating this is that anecdotal and case report evidence indicates that people who have previously changed their gender presentation (such as by transitioning) may acquire gender fluid or gender non-conforming behaviors or desires during dementia (12, 13, 19). People who have surgically transitioned may be surprised by their ‘unexpected’ body parts (12, 13). Presence or lack of familial support for transitioning or for gender fluid / gender non-conforming behavior may be particularly influential in the wellbeing of this population.

### 4.3 Gender by proxy in institutions and formal caregiving

Institutions play a key role in managing the appearance of residents with dementia, such as by regulating the type of clothes they may wear, how hair is styled, personal grooming, and providing or prohibiting items like makeup (20, 75). This is a way for institutions to communicate the quality of their care to families and auditors, rather than as a way to honor the desires and preferences of older adults (75). For example, only polyester clothes which can handle extremely high-heat wash and sanitization cycles may be allowed (20, 75).

Beyond these practicality- and rules-based ways of regulating gender presentation via appearance, the role of institutions in policing gender and sexual identity and performance may be most obvious in the experiences of LGBT+ older adults who violate the norms institutions operate on. LGBT+ older adults experience multiple forms of discrimination and normative regulation from long-term care institution staff, such as refusal to use chosen names or pronouns, exclusion or isolation by staff, refusing to acknowledge gender identity, ignoring or ‘forgetting’ a same-sex or LGBT partner designated as the medical power of attorney, refusing to admit or re-admit the LGBT older adult, and requiring that intimate care tasks (bathing, dressing) be performed by someone of the same sex as the resident’s sex assigned at birth (81, 82). These acts implicate the broader social rules that some institutions enforce on their residents in maintaining ‘appropriate’ gender and sexual identity performances.

Even institutions that are empathetic and open to providing personalized gender care may be ill-equipped to respond to changes in gender presentation or identity. As an example, consider the experience of Jamie, a trans woman who was diagnosed with dementia:

Jamie [was] a 94-year-old trans woman in Canada, who had been living openly as a woman since the age of 80 …

Jamie was diagnosed with dementia and admitted to a residential care facility for older people after Jamie’s wife died. Jamie had been taking oestrogen and steroids, but this was discontinued upon her admission for unknown reasons. Upon admission, Jamie was referred to a psychiatrist because of apparent gender confusion: ‘She was confused as to whether she was male or female, asking, “What am I?” She frequently looked down at her breasts and asked, “Where did these come from?”’ (p. 113). Jamie sometimes expressed a wish to live as a man and sometimes as a woman. Jamie would ask to wear men’s clothing, but would then point to items of women’s clothing in the wardrobe. The staff did not know how to respond: ‘What pronoun should they use? Should her clothing and haircut be feminine or masculine?’ (p. 113). Jamie’s daughter, who visited (removing ‘some of the more effeminate clothing’ from Jamie’s closet, p. 113) did not accept Jamie’s (now apparently ambivalent) gender identity as a woman: ‘She [his daughter] stated, “He’s putting aside the façade that he had been maintaining. … He never really was a transsexual, because he never had the surgery and physically he is male”’ (p. 114). The psychiatric assessment concluded that Jamie’s history was ‘consistent with a diagnosis of Gender Dysphoria-Posttransition according to the DSM-5′ (American Psychiatric Association, 2013: 116).

The assessment also concluded that Jamie exhibited ‘considerable gender identity ambivalence’. In terms of how the staff should respond to this ambivalence, the most obvious solution, that they should respond to Jamie’s presentation at any given moment in time, and not try to get Jamie to conform to ‘either/or’ binary notions of gender, does not appear to have been considered. Instead, the possibility of a ‘gender neutral’ approach had been suggested, whereby ‘staff would avoid the use of pronouns and dress Jamie in a gender neutral manner’ (p. 116). However, because it was thought that Jamie’s co-residents would find this difficult, resulting in further ostracism, it was decided that a (gender) ‘choice had to be made’ on Jamie’s behalf. Jamie died before such a choice was made (12).

Jamie’s story illustrates that even though the long-term care facility was willing to assist Jamie in presenting the way she / he / they wanted, there were still difficulties in gaining support from family members (Jamie’s daughter) and other residents. The staff decided they needed to make a ‘choice’ ‘on Jamie’s behalf’, or to determine which gender should be chosen to perform by proxy. The staff had no solid idea on what grounds to make such a choice, nor did they have training on what ‘responding to Jamie’s presentation at any given moment’ would look like. Because of this, they resorted to binarist thinking, in which one gender and one gender only would be chosen for Jamie.

### 4.4 Dementia and personal transformation

Dementia can “inspire both personally and cognitively transformative experiences,” including gender performance and/or identity (77). This has been frequently pathologized within literature that describes it (13, 19, 83). For example, Marshall et al. describe an older transgender person with dementia who developed gender fluid behaviors as having “gender confusion” and that they were “no longer able to express a consistent gender preference due to moderate dementia” (83). This approach has been critiqued as being stigmatizing and pathologizing of both changes in cognition and gender non-conforming or gender fluid behavior and/or identity, as well as playing into long-standing narratives that transgender and gender non-conforming people are simply confused or are not really the gender they say they are (13, 19).

Baril and Silverman (13) argue in favor of an age- and disability-positive approach that supports fluid gender presentation for older adults with dementia. While they focus on trans and gender non-conforming older adults, I extend this to all older adults who may change the way they engage in gendered grooming and dress for a variety of reasons, from comfort to a new identity to exploration. Baril and Silverman argue that people with dementia can be supported in an embodied selfhood that is affirming, similar to other aspects of person-centered care for dementia (13, 67–69). Dementia can reduce the perceived importance of social norms and expectations, such that “the experience of dementia may actually generate an emancipatory space in which to explore hidden, forgotten, or quite new aspects of self and identity in ways that have not previously been possible” (12). Of course this will not always be possible; many people with dementia will have a diminished quality of life and relationship to their self that is not emancipatory or based in personal exploration. But maintaining an openness and willingness to affirm the experiences that are positive can be extremely beneficial.

### 4.5 Doing gender by proxy for others

Conceptually exploring gender performance within dementia also reveals the extent to which maintaining a gendered performance is partially (and sometimes wholly) for the benefit of others, rather than for the person with dementia. The importance of gender may diminish for a person with dementia, but maintaining the appearance of a gendered performance continues to be important for the family members, friends, caregivers, and institutions surrounding the person. This highlights the relational and interactive nature of gender performances, as well as gender’s centrality to our perception of others as human (14, 15, 24). Jamie’s care staff felt that other residents would be more welcoming and accepting if Jamie had a stable gender identity, pronouns, and appearance (12). Making a ‘choice’ about which gender to perform on Jamie’s behalf was not for Jamie’s benefit, but for the people and institutions interacting with Jamie (12, 15). While doing gender by proxy can be an important way to maintain the autonomy and personal identity of the person with dementia, there is also the potential to do gender by proxy in a way that prioritizes the needs and desires of other people over the needs and desires of the person with dementia. This has the potential to do harm when it limits the autonomy or disrespects the preferences (current or former) of the person with dementia.

## 5 Discussion

This conceptual analysis has reviewed gender performativity theory, bioethical theories of surrogate decision making, and how gender is done by proxy through surrogate decision making of both informal and formal caregivers of people with dementia. As people with dementia progressively lose the ability to act independently, their caregivers must make decisions about their dress, grooming, personal care, activities, as well as how to talk to and about them and even arrange circumstances to help them feel a particular way (5, 11, 22). This process can be understood as caregivers doing gender by proxy, or performing gender on behalf of the person with dementia to help them maintain their identity and personhood (9, 16). Empirical work is needed to validate and explore this concept.

Current literature indicates that despite gender being an integral part of social relationships and embodied selfhood, little consideration is given to how people with dementia experience gender. Gender is not commonly considered in end-of-life care planning; if appearance care is considered when making end-of-life plans, the possibility for changes in gender presentation is rarely considered except by older adults who are trans, nonbinary, or gender-nonconforming. However, just as with medical care, the gender preferences, desires, and values of people with dementia may change as the disease progresses, leaving caregivers with uncertainty as to whether to honor the person’s values and preferences from before dementia or to support their current values and preferences (4, 9, 11, 13, 64). While advance care planning may provide some indications of how an older adult would like to be treated in their end of life, care planning should include enough flexibility for the surrogate or supportive decision maker to respond to emerging preferences and desires for older adults with dementia who may change their minds.

In institutional settings, a dramatic expansion is needed in training to prevent the ongoing discrimination and mistreatment against LGBT+ older adults (81, 82). It seems likely, though needs to be investigated more thoroughly, that older adults who do not explicitly identify as LGBT+ but display gender non-conforming behaviors or desires may be similarly mistreated; if so, this needs to be definitively stopped. All older adults deserve supportive and affirming care that prioritizes their psychological, emotional, and physical wellbeing and comfort.

### 5.1 Limitations

This analysis is purely conceptual, and while the literature and examples are indicative, significant research is needed to empirically validate the concept of doing gender by proxy. Furthermore, the literature included is representative of Western and particularly Anglophone theoretical traditions. Whether the concept of doing gender by proxy would hold in non-Western settings cannot be determined until further research is conducted.

### 5.2 Future directions for research

Future research on changing gender presentation is needed for older adults with dementia and their caregivers. Current research on gender experiences in older adults with dementia focuses heavily on people who identify as LGBT+ prior to their dementia diagnosis (13, 19, 83), with some exceptions, such as Sandberg’s *Dementia and the gender trouble* (9). However, changes in the relationship to and importance of gender vary for people of all gender identities; cisgender people have a relationship to gender and, like Diane, may change their gender performance even if they do not change their gender identity.

First, both qualitative and quantitative research is needed into gender experiences of people with dementia. Older adults are commonly conceptualized of as being de-gendered, even if that does not align with their identity, experience, or desires (9, 19, 20). Therefore, older adults with dementia (particularly in institutional settings) may have limited control over what they wear or how they are groomed, and providing them the opportunity to explore what will make them comfortable and feel an embodied connection to their gender could help maintain their sense of autonomy and personhood (9, 13). Quantitative research can establish the frequency and prevalence of gender non-conforming behaviors among older adults with dementia, and qualitative research can investigate the meaning-making around gender behaviors and experiences among this population. Additionally, how gender is experienced during dementia should be qualitatively explored. Even if a person does not engage in any changes in their gender presentation, they may have changes in how they relate to or feel about their gender (22, 43, 44).

Second, the needs of community and informal caregivers for people with dementia who have unexpected changes in gender performance or identity should be determined. It is unclear how prepared caregivers feel to respond to these situations in a way that affirms and supports the experience of the person with dementia and does not further pathologize them or their experience of gender. Qualitative studies among caregivers and individuals with dementia can indicate what types of support they need as they adapt to doing gender by proxy for the person they care for (if indeed this is occurring). Understanding the different needs of cisgender / heterosexual and LGBT+ caregivers in this area is critical.

Finally, institutional settings seem to range from actively hostile to unprepared to help older adults who are LGBT+ or who experience new changes to their gender performance or identity (19, 21, 79, 81, 82). Thus, finding ways to change the culture of long-term care institutions and improve the quality of gender care for all older adults is extremely important. Ethnographic studies which examine the current practices of institutional settings in regulating the gender performances of older adults with dementia, or among older adults broadly, would be extremely useful in providing evidence for changes to policy and practice which would improve the experience of older adults.

Gender is integral to the embodied sense of selfhood and personal identity of almost all people. Making space for an affirming and person-centered response to older adults with dementia can help them maintain a positive connection to their body and sense of self.

## Data Availability

The original contributions presented in the study are included in the article/supplementary material, further inquiries can be directed to the corresponding author.
